# Reply to: PREDICT underestimates survival of patients with HER2-positive early-stage breast cancer

**DOI:** 10.1038/s41523-023-00515-4

**Published:** 2023-03-16

**Authors:** Elisa Agostinetto, Evandro de Azambuja, Matteo Lambertini

**Affiliations:** 1 Institut Jules Bordet and l’Université Libre de Bruxelles, Brussels, Belgium; 2 Department of Internal Medicine and Medical Specialties (DiMI), School of Medicine, University of Genova, Genova, Italy; 3 Department of Medical Oncology, UOC Clinica di Oncologia Medica, IRCCS Ospedale Policlinico San Martino, Genova, Italy

**Keywords:** Breast cancer, Prognosis

**replying to** Ahmed M. Alaa et al. *npj Breast Cancer* 10.1038/s41523-023-00514-5 (2023)

Refining prognostication in patients with early breast cancer represents one of the main challenges of modern oncology. In our prior analysis within the phase III ALTTO trial, PREDICT showed to underestimate survival of patients with HER2-positive early breast cancer. Alaa and colleagues tested PREDICT in patients from the UK National Cancer Registry. Consistently with our findings, PREDICT highly underestimated the survival of patients with HER2-positive early breast cancer. In this manuscript, we compare the findings of both studies, and we discuss the future perspective of prognostication in breast cancer.

We read with great interest the *Matters Arising* manuscript by Alaa and colleagues^1^, in which the authors present a new prognostic model guided by artificial intelligence to improve the accuracy of survival prediction in patients with early breast cancer.

In their study, Alaa and colleagues analysed the data of 395,862 patients extracted from the UK National Cancer Registration and Analysis Service (NCRAS)^1^. First, they conducted a validation experiment to test the prognostic performance of PREDICT in this cohort; secondly, they tested a new artificial intelligence-based tool (i.e., *Adjutorium*, available at https://adjutorium-breastcancer.herokuapp.com/) to evaluate its prognostic performance in the same cohort^1^.

The first part of their study, namely the validation of PREDICT in the NCRAS cohort, allows an indirect comparison with our previously published findings^2^, in which we evaluated the prognostic performance of PREDICT in patients with HER2-positive early breast cancer enrolled in the phase III, randomized ALTTO trial^3^.

Both our results and those from Alaa and colleagues were consistent in showing that PREDICT underestimates the survival of patients with HER2-positive early breast cancer. When observing the magnitude of this underestimation, we found an absolute difference in 5-year overall survival of −6.69% (95% confidence interval −7.55 to –5.83), compared to −16% in the study by Alaa et al. (Table 1). This difference in the magnitude of underestimation could be attributed to the different population analysed. The cohort evaluated by Alaa and colleagues consists in a large real-world population, with diagnoses of breast cancer ranging from 2000 and 2016, and likely heterogeneous in terms of treatments received. On the contrary, our cohort from the ALTTO trials included patients randomized in a phase III trial enrolling between June 2007 and July 2011.

**Table 1:** Magnitude of survival underestimation by PREDICT in the study by Agostinetto et al. and in the one by Alaa et al.

	Agostinetto et al.	Alaa et al.
Study population	Patients with HER2-positive early breast cancer enrolled in the ALTTO trial	Real-world patients with early breast cancer extracted from the UK NCRAS registry
*N*	2794	395,862
Enrollment years	2007–2011	2000–2016
Underestimation of 5-year OS by PREDICT in HER2-positive early breast cancer	7%	16%
Underestimation of 5-year OS by PREDICT in HER2-negative early breast cancer	NA	1%

*ALTTO* Adjuvant Lapatinib and/or Trastuzumab Treatment Optimization Trial, *NCRAS* National Cancer Registration and Analysis Service, *NA* not applicable

On the other hand, some differences exist between our study and the one from Alaa and colleagues. First, they evaluated the prognostic performance of PREDICT also in patients with HER2-negative breast cancer (analysis not performed in our study, that included only patients with HER2-positive breast cancer). In HER2-negative breast cancer, PREDICT underestimated survival by 1% only. This finding may suggest that PREDICT still has a role in hormone receptor-positive breast cancers, i.e., the subtype for which this tool was originally developed.

Alaa and colleagues also tested PREDICT in patients with estrogen receptor (ER) positive breast cancer from the NCRAS registry data, apparently regardless of HER2-status. In this sub-cohort, PREDICT underestimated survival. Nonetheless, we believe that it would be more informative to add further granularity in this analysis, by evaluating separately ER-positive/HER2-negative vs. ER-positive/HER2-positive vs. ER-negative/HER2-positive tumours. Of note, these subgroups are associated with different clinical-pathological and molecular characteristics^4^, and merging all together could easily results in biases. Going even further, additional attention to more specific subgroups, like ER-low^5^ and HER2-low^6^ breast cancers, is getting increasing interest and could provide interesting insights.

Furthermore, Alaa and colleagues presented a new prognostic model, *Adjutorium*, based on artificial intelligence, to improve the accuracy of survival prediction in patients with early breast cancer. *Adjutorium* was associated with better survival estimation in all the analyzed sub-cohorts (i.e., HER2-positive and ER-positive). In a prior publication by the same team, *Adjutorium* was compared to PREDICT and showed improved discriminatory accuracy^7^. These data are encouraging, and support further investigation of this tool. Hence, we fully support the interest of Alaa and colleagues in testing this model in patients enrolled in the ALTTO trial.

All these research efforts underline the relevance of prognostication in patients with early breast cancer. Traditionally, this was considered as an important topic especially for patients with hormone receptor-positive/HER2-negative disease, for whom prognostication could affect the choice of adding adjuvant chemotherapy before endocrine therapy initiation as well as for extended duration of endocrine therapy (Table 2)^8,9^. However, also in HER2-positive disease prognostication has paramount importance. Indeed, although most patients receive (neo)adjuvant chemotherapy as per current standard of care, prognostication can still impact crucial aspects of patient’s life, including for instance family planning in premenopausal women^10^. Moreover, an increasing number of clinical trials are testing de-escalation and escalation treatment strategies to optimize the therapeutic approach of patients with all subtypes of breast cancer. It is likely that in the future there will be an increasing incorporation of molecular information to provide reliable prognostic estimates^11^. Nevertheless, online tools like *Adjutorium* retain the strengths to be free, publicly available, fast, and easy-to-use in daily clinical practice.

**Table 2:** Prognostic models for patients with early breast cancer.

Name	Type of information analyzed	Information analyzed	Recommendation by ASCO guidelines	
			ER + /HER2-	HER2 + (ER + or ER-)
Oncotype-DX	Genomic	21 genes	—Pre and post-menopausal patients with node-negative disease (high/strong) —Postmenopausal patients with 1–3 positive nodes (high/strong)	No mature evidence to recommend use in this population
Mammaprint	Genomic	70 genes	—Postmenopausal patients with high clinical risk and node-negative disease (intermediate/strong) —Postmenopausal patients with high clinical risk and 1–3 positive nodes (intermediate/strong)	No mature evidence to recommend use in this population
EndoPredict	Genomic + Clinical	12 genes + tumor size and nodal status	—Postmenopausal patients with node-negative disease (intermediate/moderate) —Postmenopausal patients with 1–3 positive nodes (intermediate/moderate)	No mature evidence to recommend use in this population
Prosigna	Genomic + Clinical	Intrinsic subtype (PAM50) + proliferation genes + Tumor size + nodal status	—Postmenopausal patients with node-negative disease (intermediate/moderate)	No mature evidence to recommend use in this population
Breast Cancer Index (BCI)	Genomic	HOXB13:IL17BR (H/I) ratio Molecular Grade Index (MGI)	—Postmenopausal patients with node-negative disease (intermediate/moderate) —Postmenopausal patients with 1–3 positive nodes (intermediate/moderate) —Patients who received 5 years of endocrine therapy	No mature evidence to recommend use in this population
RSClin	Genomic + Clinical	21 genes (i.e., Oncotype RS) + age, tumor size, and grade	No specific recommendations so far	No mature evidence to recommend use in this population
IHC4	Clinical	ER, PgR, HER2, Ki67	—Postmenopausal patients with node-negative disease (intermediate/moderate)* —Postmenopausal patients with 1–3 positive nodes (intermediate/moderate)* *only if locally validated and in case of no access to genomic tests.	No mature evidence to recommend use in this population
PREDICT	Clinical	Age, menopausal status, ER, HER2, Ki67, tumor size, nodal status, grade, detection modality, treatments	No specific recommendations so far	No mature evidence to recommend use in this population
CTS5	Clinical	Age, tumor size, grade, nodal status	May be used in patients who received 5 years of endocrine therapy	No mature evidence to recommend use in this population
Regan risk score	Clinical	Age, nodal status, tumor size, grade, ER, PgR, Ki67	No specific recommendations so far	No mature evidence to recommend use in this population
Adjutorium	Clinical	Age, tumor size, nodal status, grade, detection mode, ER, HER2, treatments	No specific recommendations so far	No mature evidence to recommend use in this population

In the rapidly changing treatment landscape for early breast cancer, the challenge of prognostic models is to allow a promptly adaptation to the use of new therapies and to the subsequent changes in patients’ outcomes.

## Reporting Summary

Further information on research design is available in the Nature Portfolio Reporting Summary linked to this article.

## Supplementary information

Reporting Summary
